# The effects on population health status of using dedicated property taxes to fund local public health agencies

**DOI:** 10.1186/1471-2458-11-471

**Published:** 2011-06-14

**Authors:** Peggy A Honoré, Peter J Fos, Xueyuan Wang, Ramal Moonesinghe

**Affiliations:** 1 Department of Community Health Sciences, University of Southern Mississippi, 118 College Drive, Hattiesburg, MS 39406, USA; 2 Health Policy and Systems Management Program, School of Public Health, Louisiana State University Health Sciences Center, 2020 Gravier Street, New Orleans, LA 70112, USA; 3 Office of Minority Health and Health Disparities, Centers for Disease Control and Prevention, 1600 Clifton Road, Atlanta, GA 30333, USA

## Abstract

**Background:**

In the United States, a dedicated property tax describes the legal authority given to a local jurisdiction to levy and collect a tax for a specific purpose. We investigated for an association of locally dedicated property taxes to fund local public health agencies and improved health status in the eight states designated as the Mississippi Delta Region.

**Methods:**

We analyzed the difference in health outcomes of counties with and without a dedicated public health tax after adjusting for a set of control variables using regression models for county level data from 720 counties of the Mississippi Delta Region.

**Results:**

Levying a dedicated public health tax for counties with per capita income above $28,000 is associated with improved health outcomes of those counties when compared to counties without a dedicated property tax for public health. Alternatively, levying a dedicated property tax in counties with lower per capita income is associated with poor health outcomes.

**Conclusions:**

There are both positive and negative consequences of using dedicated property taxes to fund public health. Policymakers should carefully examine both the positive association of improved health outcomes and negative impact of taxation on poor populations before authorizing the use of dedicated local property tax levies to fund public health agencies.

## Background

In economic terms, public health is a public good. It is consumed by everyone collectively and benefits all of society equally. Safe drinking water, control of epidemics, and reducing the risk of environmental hazards all illustrate the collective consumption and societal benefits of public health. The public health role, at all levels of government, for global disease surveillance, distribution of pharmaceuticals, and public messaging to reduce the spread of H1N1 throughout the world is a timely example. Passage of the Patient Protection and Affordable Care Act (ACA) provides additional illustration. Authority in ACA for national investments in a Prevention and Public Health Fund is intended to generate health benefits throughout all sectors of society [1].

Every community in America is served by a local public health agency [2] and everyone in those communities benefit from public health services whether they pay for it or not. In public health, as with other functions classified as public goods (e.g., law enforcement, public education, sanitation, fire protection), it is not possible to assign specific costs or charge for additional users who receive the benefits from these public services [3].

The public financing role of government is to allocate resources to meet these critical public-sector needs of society that are not fulfilled sufficiently by private markets. Because public health services are public goods, it is financed primarily through the allocation of government resources. Similar to other public services, local public health functions are funded by local governments and supplemented by state and federal revenues. In 2008, local public health agencies were funded 25% from local revenues, 20% from states, 17% from federal sources, with the remaining balance from Medicaid and Medicare reimbursements and other sources [4]. Examining the extent that dedicated property taxation is used as a source of local revenues to finance public health and the association of those investments to population health outcomes is the focus of this paper.

### Taxation

The primary source of revenue for local governments is taxation. The most prevalent form of local taxation is property taxes. In 2005, tax revenue ($484 billion) represented 63% of all local revenues [5]. Local property taxes ($347 billion) accounted for 45% of all local revenues and 72% of all local tax revenue [5]. In 2007, local property tax collections per capita in the United States was $1,236, up 13% from 2005 [6].

The origin of property taxation in the United States dates back to the 17^th ^century [7]. Local government authority to levy and collect property taxes is granted by state constitutions and statutes [8,9]. The 10^th ^Amendment to the United States Constitution is where power is reserved for states to grant authority to local jurisdictions to levy and collect local property taxes. The "*benefit view" *theory is used by some economists to characterize the property tax as a charge to residents for local public services such as education and fire protection [10].

A *dedicated *property tax describes the legal authority to levy a tax millage rate against assessed property value for a specified purpose and distributing the tax collections as prescribed in the law. As an example, over half of all property tax collections in the United States are dedicated to K-12 education [7]. Alternatively, property tax revenues are also commonly accumulated in a general (non-specified) fund. Local authorities typically decide annually how to allocate general fund tax revenues to specific public purposes. A dedicated tax millage rate can safeguard public entities against local revenue allocation fluctuations that may occur during annual budget negotiations with government officials.

In addition to the traditional public-sector enterprises that utilize the dedicated property tax system (e.g., hospitals, libraries, museums, parks, cemeteries, animal control, sewer systems, harbors) others are beginning to use this method to ensure a defined source of local revenues. As an example, five states, Ohio, Louisiana, Kansas, North Dakota, and Michigan, now levy a local dedicated property tax to fund senior services [11]. Ohio collections in 2006 were $200 million and funded senior programs such as transportation, in-home services, case management, and nutrition [11].

### Return on Taxpayer Investments

Some problems drive the formulation of policies more than others [12]. When combined with a sense of urgency, health problems can shape public opinion in favor of new policies. Allocation policies, such as taxation, are commonly used as subsidies to ensure the supply of services and to meet public objectives [12]. However, maintaining support for taxation policies can be greatly influenced by demonstrating a return on taxpayer investments. The public simply expects that a tax be justified with economic benefits [13]. Documenting benefits should be a routine strategy to garner and maintain support. The example below is provided to illustrate this point.

While healthcare in America for the most part is treated as a commodity, some sectors of healthcare do serve a public good and, as such, are funded with tax revenues. Community-based public hospitals are a classic example. The inability to sustain certain healthcare services with private financing may necessitate that tax revenues be used to assure the delivery of care for residents. Special hospital taxing districts are formed throughout the United States for such purposes and have authority to levy dedicated taxes to finance operations in these organizations. Research by Studnicki et al. showed that jurisdictions in Florida that use this form of financing for public hospitals experienced better community level health outcomes when compared to state means [14]. These findings provided evidence on the impact of the special taxing districts when the taxing policies were under critical review. Monitoring to assure that these healthcare services are available in communities is a traditional role of public health.

## Methods

### Study Purpose and Population

In this cross-sectional study we investigated to determine if the use of dedicated property taxation to finance local public health agencies was associated with improved population health status. Taxation is used to redistribute resources. The ability to show benefits received from such redistributions is important for demonstrating the value and return on those taxpayer investments for public health services.

The population examined in this research represents 720 counties in eight states known as the Mississippi Delta Region (Alabama, Arkansas, Illinois, Kentucky, Louisiana, Mississippi, Missouri and Tennessee). Of the 720 counties in these states, 240 share a national designation as a Delta county because of common characteristics in population health status and socioeconomic conditions.

### Data Collection

We collected county level data on the utilization of dedicated property tax levies to fund local public health agencies (i.e., services and facilities) in 720 counties of the eight states in the Mississippi Delta Region. Based on the U.S. 2000 Census, total population for the region was estimated to be 42 million. This study covered the 3-year period 2003-2005.

An initial step was to categorize the 720 counties as those that did or did not levy a dedicated tax for public health services. If a local government levied a tax dedicated for the local public health agency during the study period 2003-2005, we defined that county in this research as a county *with *a public health tax. There were a few instances where a county did not levy a tax for public health but a large city in that county did levy a tax dedicated for public health. If a city, with a population that represented more than 60% of the total county population, had a dedicated tax levy for the city public health agency, we considered that county as a county *with *a public health tax even if the county had not levied a tax for the local public health agency. For example, Green County and Buchanan County in Missouri were both considered counties *with *a public health tax because the City of Springfield in Green County and the City of St. Joseph in Buchanan County had city tax levies dedicated for the city public health agencies. The population in both of those cities represented over 60% of the total county population. Given the population size of those cities, we considered any potential impact of that tax to have influence on the majority of the county's population.

The legislative period for the property tax millage rate can vary from state to state, but is typically for multiple periods (i.e., 5 to 10 years). For any reason, if a tax levy dedicated for a county public health agency was not levied continuously during the study period, that county was considered a county *without *a public health tax. Based on these criteria, there were 338 counties *with *a public health tax and 382 counties *without *a public health tax in the study population. Fifty percent (n = 239) of the 480 non-Delta counties had a dedicated public health tax during the study period whereas only 41% (n = 99) of the 240 Delta counties had a dedicated public health tax. None of the counties in Arkansas, Mississippi, and Tennessee had a tax levy dedicated to public health agencies. Mississippi counties have authority to levy a dedicated tax for public health but Arkansas and Tennessee do not have such authority.

### Outcome Measures

County-level data that are typically used as measures of community health status were collected for a set of health variables available from national, state, and local datasets. Invasive cancer incidence rates for each county were obtained from state cancer registries or the state health department. Other health outcome measures were obtained from the CDC Wonder website [15]. We measured the health status of the counties using mortality rates for overall population, cardiovascular disease (CVD), cerebrovascular disease (stroke), heart disease, chronic lower respiratory disease (CLRD), diabetes, pneumonia/influenza, lung and bronchus cancer, all types of cancer and unintentional injury; incidence rates for lung and bronchus cancer, colorectal cancer, prostate cancer, female breast cancer and all types of cancer; and years of potential life lost rate before age 75 (YPLL75).

These health outcomes are a subset of the health outcomes studied by Studnicki et al. based on six categories: total population mortality, major disease mortality, cancer mortality and morbidity, avoidable hospitalizations, trauma/accidents mortality, and infectious diseases [14]. We excluded the categories of avoidable hospitalizations and infectious diseases due to not having county level data for these outcomes or having small number of cases leading to unstable rates at the county level.

A county is considered to have an unreliable mortality rate for a disease when the number of deaths is less than 20 over the 3-year period studied. Additional file 1 shows the outcome variables included/excluded and the percent of counties with unreliable rates in the remaining four categories. Except for pneumonia/influenza and diabetes with around 43% percent of counties having unreliable data, the rest of the health outcomes we selected had less than 18% of counties with unreliable data. When a county had unreliable data for the health outcomes we selected, we used the Indirectly Standardized Mortality Rate for that county [16].

The mortality rates, incidence rates, and rates for other outcome measures were calculated for the 3-year period 2003-2005 except for the Illinois cancer incidence rates which were for the 5-year period 2001-2005. Even if we hypothesize that the counties with a public health tax have better health outcomes compared to counties without a public health tax, there are other factors that can influence health outcomes differentially in different counties. Therefore, to adjust the difference in health outcomes between these two groups of counties due to these other factors, we used a regression analysis with a set of control variables. For control variables we selected demographic and economic variables: population size, percent net migration, percent population over 65, percent population under 18, percent non-white population, percent rural population, percent below poverty level, percent Medicaid eligible, number of physicians per 1,000 population, per capita income, unemployment rate, median household income, and whether the county is a Delta county or not. All of the control variables were expressed as annual averages during the study period except for percent rural population, which was based on year 2000 population. Population size was categorized into three groups: population less than 25,000, between 25,000 and 50,000, and over 50,000.

A preliminary analysis of the regression model indicated problems with multicollinearity due to highly correlated control variables. After removing these variables from the regression model, the control variables for the final model were population size, percent net migration, percent non-white population, percent population over 65, percent rural population, percent Medicaid eligible, number of physicians per 1,000 population, unemployment rate, per capita income, and whether the county is a Delta county or not.

### Statistical Analysis

It is well known that health outcomes are highly correlated with per capita income [17]. Plots of some outcome variables versus per capita income showed a quadratic relationship between health outcomes and per capita income. Similar trends have been seen in other studies. For example, a plot of life expectancy in the Organization for Economic Co-operation and Development (OECD) countries versus heath spending per capita shows a quadratic relationship [18]. Therefore, we used the regression model,

where E(y) is the mean value of the health outcome variable, h = 1 or 0 depending on whether the county has a public health tax or not, c is the per capita income, f is a linear combination of the control variables and b_0_, b_1_, b_2_, b_3_, b_4_, and b_5 _denote the regression coefficients of the constant term, h, c, c^2^, the interaction between h and c, and the interaction between h and c^2 ^respectively. The mean difference in health outcomes between counties with a public health tax and without a public health tax is then given by

which is a quadratic function of the per capita income. If none of the coefficients for quadratic terms in the regression were statistically significant, the variables corresponding to them were removed from the regression model and the mean difference in outcomes is linear in per capita income (E(y)|_h = 1 _- E(y)|_h = 0 _= b_1 _+ b_4_c ). When linear terms also are not statistically significant, the mean difference in outcomes is the regression coefficient of h (E(y)|_h = 1 _- E(y)|_h = 0 _= b_1_).

## Results

The mean values of health outcomes and control variables between counties with and without a public health tax are given in Table 1. The average percent minority population for counties without a public health tax is significantly higher with more than double that of counties with a public health tax. The average percent below poverty level, percent Medicaid eligible, and percent uninsured per county are significantly higher for counties without a public health tax and the average household income is significantly lower for counties without a public health tax. The average age adjusted overall mortality rate, age adjusted mortality rate for heart disease, cardiovascular disease, stroke, pneumonia/influenza, and average years of potential life lost rate are significantly higher for counties without a public health tax compared to counties with a public health tax. On the other hand, the average incidence rate for all types of cancer, lung and bronchus cancer, colorectal cancer and female breast cancer are significantly lower for counties without a public heath tax compared to counties with a public health tax.

**Table 1:** Comparisons of Counties with and without Dedicated Local Public Health Tax Levy

	Dedicated Local Public Health Tax Levy			
	**With a tax (n = 338)**	**Without a tax (n = 382)**	**t-value**	Pr > t*
**Demographic/socioeconomic**				
Mean total population	53,454	65,670	0.77	0.4389
Percent net migration (net migrants/total population)	1.32	0.71	-1.14	0.2531
Population aged >65 years (%)	14.5	14.25	-1.13	0.2568
Population aged <18 years (%)	23.94	24.34	2.21	0.0272
Non-whites of total population (%)	9.69	22.22	9.83	<.0001
Per capita Income ($)	23,920	23,485	-1.32	0.1874
Household income ($)	35,297	33,026	-3.71	0.0002
Poverty (%)	16.29	17.88	3.68	0.0002
Percent rurality (rural population/total population) ^§^	63.29	66.71	1.67	0.0951
Medicaid eligible (%)	24.07	27.7	5.87	<.0001
Physicians (total licensed MD or DO/1,000 total population)	1.1	1.18	0.99	0.3218
Unemployment (%)	6.37	6.53	1.37	0.1714
Uninsured^§ ^(%)	13.94	15.57	5.69	<.0001
**Total population mortality **(per 100,000 population)				
Age-adjusted mortality	939.74	972.83	3.88	0.0001
Years of potential life lost (before age 75)	8,828.90	9,902.10	7.13	<.0001
**Major disease mortality **(age-adjusted/100,000 population)				
Heart disease	261.14	276.98	4.21	<.0001
Cardiovascular disease (CVD)	339.45	363.75	5.91	<.0001
Cerebrovascular disease (Stroke)	56.31	63.01	5.76	<.0001
Chronic lower respiratory disease (CLRD)	52.79	51.29	-1.22	0.2222
Diabetes	29.07	29.42	0.36	0.7161
Pneumonia/influenza	25.74	27.45	2.05	0.0410
**Cancer mortality and incidence **(age-adjusted/100,000 population)				
All types of cancer mortality	218.34	214.29	-1.95	0.0518
Lung and bronchus cancer mortality	70.42	68.61	-1.50	0.1336
All types of cancer incidence	482.3	441.2	-10.23	<.0001
Lung and bronchus cancer incidence	88.78	80.18	-6.37	<.0001
Colorectal cancer incidence	58.46	53.4	-6.14	<.0001
Female breast cancer incidence	116.34	108.04	-5.28	<.0001
Prostate cancer incidence	141.84	141.04	-0.28	0.7823
**Trauma/accidents (**age-adjusted/100,000 population)				
Unintentional injury mortality	58.93	60.43	1.07	0.2857

* P-value < 0.05 indicating statistically significant

^§ ^2000 year data only

Table 2 gives the estimates of the regression coefficients and the p-values for variable h (), the interaction between h and c (), and the interaction between h and c^2 ^() when the regression coefficients are statistically significant at 0.05 significance level for all the health outcomes considered. Non-significant regression coefficients (NS) were removed from the final model.

**Table 2:** Estimates of regression coefficient and p-value for health outcomes

Health outcome						
	
	Estimate	p-value	Estimate	p-value*	Estimate	p-value
Mortality rate, population	438.25	0.0004	-29.08	0.0018	0.44	0.0105

Mortality Rate, heart disease	154.43	0.0110	-10.85	0.0186	0.17	0.0422

Incidence rate, all types of cancer	226.26	0.0036	-13.86	0.0187	0.24	0.0321

Incidence rate, lung and bronchus cancer	81.20	0.0005	-5.21	0.0032	0.08	0.0106

YPLL75	6119.20	0.0007	-453.59	0.0010	7.77	0.0025

Mortality Rate, CVD	133.70	0.0394	-9.92	0.0441	0.16	0.0747

Mortality Rate, CLRD	11.21	0.0739	-0.51	0.0490	-	-

Mortality Rate, all types of cancer	51.48	<0.0001	-1.85	<0.0001	-	-

Mortality rate, lung and bronchus cancer	17.93	0.0030	-0.70	0.0048	-	-

Mortality rate, unintentional injury	15.65	0.0131	-0.58	0.0268	-	-

Mortality rate, diabetes	12.82	0.0158	-0.460	0.0375	-	-

Mortality rate, stroke	-5.06	0.0002	-	-	-	-

Incidence rate, colorectal cancer	4.54	<0.0001	-	-	-	-

Incidence rate, prostate cancer	10.18	0.0007	-	-	-	-

Incidence rate, female breast cancer	6.29	0.0005	-	-	-	-

Mortality rate, pneumonia/influenza	-	-	-	-	-	-

There is no significant difference in mean mortality rate of pneumonia/influenza between counties with a public health tax and counties without a public health tax after adjusting for control variables. The mean incidence rates for colorectal cancer, female breast cancer, and prostate cancer are significantly higher in counties with a public health tax compared to counties without a public health tax, whereas the mortality rate for stroke is significantly lower in counties with a public health tax.

The estimated mean differences in mortality rates between counties with a public health tax and counties without a public health tax for CLRD, lung and bronchus cancer, all types of cancer, unintentional injury, and diabetes are linear functions of per capita income. Because the estimated slopes of the regression lines are negative for all these health outcomes, the difference in mean mortality rates decline with increasing per capita income.

**Figure 1:**
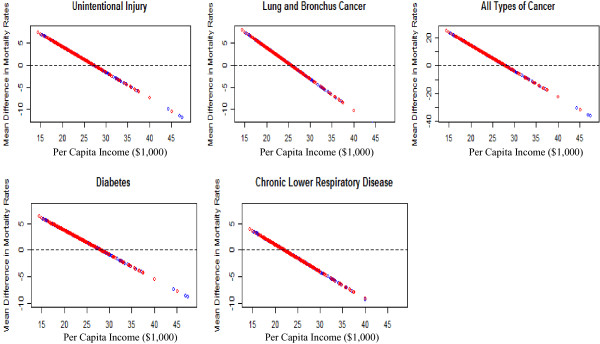
**Mortality difference between counties with and without a tax by per capita income (linear)**. Mortality rate difference between counties with and without a tax by per capita income (linear function).

Figure 1 illustrates the decline in the difference in mean mortality rates between counties with a public health tax and without a public health tax with increasing per capita income for these health outcomes. When per capita incomes are less than a range of values between $22,000 and $28,000, the mortality rates in counties with a dedicated public health tax are higher than the counties without a dedicated public health tax indicating that having a public heath tax does not improve the health outcomes in these counties. It is possible that the tax levied is a percent of the per capita income and the funds contributed to public health may be small.

**Figure 2:**
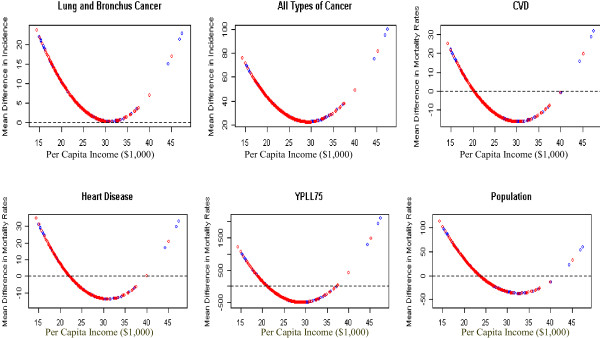
**Mortality/Incidence difference between counties with and without a tax by per capita income (quadratic)**. Mortality rate difference between counties with and without a tax by per capita income (quadratic function).

The difference in mean incidence rates between counties with a public health tax and without a public health tax for lung and bronchus cancer and all types of cancer, and the difference in mortality rates for overall population, heart disease, CVD, and YPLL75 are expressed as quadratic functions of per capita income and presented in Figure 2. The mean incidence rates for lung and bronchus cancer and all types of cancer remain higher for counties with a public health tax compared to counties without a public health tax regardless of the per capita income of the county. The mean mortality rates for CVD, heart disease, the overall population, and YPLL75 are higher for counties with a public health tax when per capita income is less than a value between $22,000 and $24,000. This indicates that levying a public health tax for counties with lower per capita income is associated with poor health outcomes. Except for four or five counties that have per capita income over $40,000 (including three counties with a public health tax), the rest of the counties with a public heath tax have lower mean mortality rates than the counties without a public health tax.

## Discussion

This paper examined the relationship between health status and dedicated taxation used for public health programming, services, and facilities. Counties that make up the Mississippi Delta Region were compared and grouped according to whether they levy a dedicated public health tax or not. Many differences were observed in this geographically homogeneous area.

Several findings resulted from this examination. Overall, income and mortality demonstrated a relationship. Specifically, the adjusted mean difference in mortality rates between counties with a dedicated public health tax and without a dedicated public health tax for lung and bronchus cancer, all types of cancer, unintentional injury, chronic lower respiratory disease, and diabetes decreased as per capita income increased. The YPLL75 is higher in counties with a public health tax and lower per capita income. It is possible that counties with low per capita income have higher mortality rates, because the underlying causes of death are exacerbated by levels of taxation on the poor.

Interestingly, levying a dedicated public heath tax for counties with low per capita income was associated with poor health outcomes. Alternatively, levying a dedicated public health tax for counties with per capita income more than $28,000 is related to better health outcomes of these counties compared to counties without a dedicated public health tax. The mortality rates for most of the diseases are higher for counties with a public health tax when per capita income is low but are lower when per capita income is high. This finding of the impact of income can be explained, in part, by the overwhelming effect of wealth on health outcomes. Income is thought to allow people to buy needed health care services. It has been found that people with low income are sicker more often and die prematurely [17]. It is known that a person's health is often poorer in a population with a low average per capita income in spite of the person's own income [17]. It is also known that as income decreases, a person's health declines, and vice versa [19]. In this study the average percent of minorities in counties without a dedicated public health tax is significantly higher than that of counties with a dedicated public health tax. Additionally, the average percent of people below poverty level, percent Medicaid eligible people, and percent uninsured people are all higher for counties without a dedicated public health tax. This follows what is expected with respect to the relationship between health and income. Further evidence is that the average household income per county is significantly lower for counties without a dedicated public health tax.

If incidence rates of disease are evaluated another interesting finding is seen. The mean incidence rates for lung and bronchus cancer and all types of cancer are higher in counties with a dedicated public health tax. This relationship is seen independent of the per capita income of each county. Overall, our results suggest that the mean incidence rates are higher, but mortality rates are lower for counties with a dedicated public health tax after adjusting for control variables that may indicate a higher rate of detection of diseases due to increased screening. For example, North America ranked highest in terms of incidence, but 9th for mortality for prostate cancer in a study of 19 worldwide regions in 2002 [20]. The widespread use of the Prostate Specific Antigen (PSA) blood testing in the U.S. resulted in not only higher prostate cancer incidence, but also a much higher proportion of early stage cases being diagnosed than in countries with lower level of testing that may have led to higher survival rates overall and lower mortality rates relative to incidence [20].

### Study Limitations

Our study is limited by not considering the overall property tax dedicated to public health. We did not adjust our results for state and federal funding of public health and had no measures to compare the performance, effectiveness and efficiency of public health system delivery of core functions in counties with and without a dedicated public health tax. We selected all of the counties in the Mississippi Delta Region for the study. Since these counties are not a random sample of counties in the U.S., our results may not be applicable nationally. Our study is a cross-sectional analysis, therefore, it is not strong in showing cause-effect relations. A related limitation in cross-sectional analysis is policy endogeneity. Because we use cross-sectional data analysis, we cannot conclude whether dedicated public health taxes influence health outcomes or if health outcomes influence the likelihood of a county levying a dedicated property tax for public health.

### Policy Implications

Debates regarding the roles and responsibilities of health policy at the different levels of government can be traced to the founding of America [12]. Taxation policy debates have endured over time as well. In the 19^th ^century, E.R.A. Seligman, a tax economist, proclaimed the property tax as "one of the worst taxes known in the civilized world" [[13] p 4]. Such arguments against local taxes persist today [21]. However, when local taxes are used they typically reflect the priorities of the local community.

Historically, examining the role and responsibility of local government for health policy has lagged behind that of federal and state government. Also, public health has not used property tax policies to the degree that these taxes are used by the nation's school systems or other public service enterprises. Generally, the public is also not widely familiar with the mission or value of the nation's public health system. Advancing local property tax policies could be a strategy for increasing the role of local government in population health improvements, while also garnering greater local support and engagement in public health. Such policies would have synergy with authority in ACA for investments in community-based public health programs as a means of improving population health [1].

## Conclusion

The Mississippi Delta Region states share common characteristics in health and socioeconomic status. However, jurisdictions within these states do not all choose to fund public health services with a locally dedicated property tax. There may be some underlying factors that contribute to the utilization of this form of financing that was not examined in this research. Findings suggest that levying a dedicated tax was associated with better health outcomes. However, when considering locally dedicated taxation, policymakers should carefully examine the local tax base and potential impact of levying a tax on poor communities.

## Competing interest

The authors declare that they have no competing interests.

## Authors' contributions

PH designed the study, served as the taxation expert, and led the writing of the manuscript. RM designed the statistical analysis and wrote the methods and results sections. XW collected and analyzed the data and assisted in writing the manuscript. PF assisted in writing the manuscript discussion section. All authors read and approved the final manuscript.

## Pre-publication history

The pre-publication history for this paper can be accessed here:

http://www.biomedcentral.com/1471-2458/11/471/prepub

## Supplementary Material

Additional file 1 **Selected Health Outcomes for Regression**. This file provides a table of health outcome variables included/excluded in the study and the number and percent of counties with unreliable rates. Click here for file
