# Supplementary material for: The effects on population health status of using dedicated property taxes to fund local public health agencies
Source: BMC Public Health. 2011 Jun 14;11:471. doi: 10.1186/1471-2458-11-471 (PMC3141454; doi:10.1186/1471-2458-11-471)
Supplement: Additional file 1 — Selected Health Outcomes for Regression. This file provides a table of health outcome variables included/excluded in the study and the number and percent of counties with unreliable rates. [file 1471-2458-11-471-S1.DOC]

Selected Health Outcomes for Regression

| **Variable** | **Number of County with Unreliable Rate** | **Percent of County with Unreliable Rate** | **Variable Selected** |
| --- | --- | --- | --- |
| **Total Population Mortality** (per 100, 000 population) |  |  |  |
| Age-adjusted mortality | 0 | 0 | yes |
| Years of Potential Life Lost rate (before 75) | 0 | 0 | yes |
| ( 2 total population mortality rates selected) |  |  |  |
| **Major Disease Mortality** (per 100, 000 population) |  |  |  |
| Cardiovascular disease (CVD) | 1 | 0.14 | Yes |
| Heart disease | 2 | 0.28 | Yes |
| Cerebrovascular disease (Stroke) | 89 | 12.39 | Yes |
| Chronic lower respiratory disease (CLRD) | 127 | 17.6 | Yes |
| Diabetes | 313 | 43.5 | Yes |
| Liver disease | 547 | 75.97 | No |
| Pneumonia/influenza | 309 | 42.92 | Yes |
| (6 major diseases mortality rates selected) |  |  |  |
| **Cancer Mortality** (per 100, 000 population) |  |  |  |
| All types of cancer | 1 | 0.14 | Yes |
| Colorectal cancer | 410 | 56.94 | No |
| Lung and bronchus cancer | 68 | 9.44 | Yes |
| Female breast cancer | 517 | 71.81 | No |
| Prostate cancer | 593 | 82.36 | No |
| (2 cancer mortality rates selected) |  |  |  |
|  |  |  |  |
|  |  |  |  |

**Selected Health Outcomes for Regression (continued)**

| **Trauma/Accidents Mortality** (per 100, 000 population) |  |  |  |
| --- | --- | --- | --- |
| Unintentional injury | 93 | 12.92 | yes |
| Motor vehicle accident | 316 | 43.89 | No |
| Intentional injury | 471 | 65.42 | No |
| Homicide | 652 | 90.56 | No |
| Suicide | 550 | 76.39 | No |
| Poisoning fatalities | 582 | 80.83 | No |
| Firearm fatalities | 540 | 75 | No |
| (1 trauma/accident mortality rate selected) |  |  |  |
| **Cancer Incidence*** (new cases of invasive cancer per 100, 000 population) |  |  |  |
| All types of cancer | 1 | 0.14 | yes |
| Colon and rectum cancer | 35 | 4.86 | yes |
| Lung and bronchus cancer | 15 | 2.08 | yes |
| Female breast cancer | 45 | 6.25 | yes |
| Prostate cancer | 37 | 5.14 | yes |
| (5 cancer incidence rates selected) |  | |  |
| Notes: Rates for cases less than 20 over 3-year period 2003-2005 were considered unreliable. | | | |
| * Illinois cancer incidence data were 5-year period 2001-2005. | | | |
